# β-Diversity and Species Accumulation in Antarctic Coastal Benthos: Influence of Habitat, Distance and Productivity on Ecological Connectivity

**DOI:** 10.1371/journal.pone.0011899

**Published:** 2010-07-30

**Authors:** Simon F. Thrush, Judi E. Hewitt, Vonda J. Cummings, Alf Norkko, Mariachiara Chiantore

**Affiliations:** 1 National Institute of Water and Atmospheric Research, Hamilton, New Zealand; 2 DipTeRis, Università di Genova, Genoa, Italy; 3 National Institute of Water and Atmospheric Research, Wellington, New Zealand; 4 Marine Research Centre, Finnish Environment Institute (SYKE), Helsinki, Finland; 5 Department of Marine Ecology – Kristineberg, University of Gothenburg, Fiskebäckskil, Sweden; University of California Davis, United States of America

## Abstract

High Antarctic coastal marine environments are comparatively pristine with strong environmental gradients, which make them important places to investigate biodiversity relationships. Defining how different environmental features contribute to shifts in β-diversity is especially important as these shifts reflect both spatio-temporal variations in species richness and the degree of ecological separation between local and regional species pools. We used complementary techniques (species accumulation models, multivariate variance partitioning and generalized linear models) to assess how the roles of productivity, bio-physical habitat heterogeneity and connectivity change with spatial scales from metres to 100's of km. Our results demonstrated that the relative importance of specific processes influencing species accumulation and β–diversity changed with increasing spatial scale, and that patterns were never driven by only one factor. Bio-physical habitat heterogeneity had a strong influence on β-diversity at scales <290 km, while the effects of productivity were low and significant only at scales >40 km. Our analysis supports the emphasis on the analysis of diversity relationships across multiple spatial scales and highlights the unequal connectivity of individual sites to the regional species pool. This has important implications for resilience to habitat loss and community homogenisation, especially for Antarctic benthic communities where rates of recovery from disturbance are slow, there is a high ratio of poor-dispersing and brooding species, and high biogenic habitat heterogeneity and spatio-temporal variability in primary production make the system vulnerable to disturbance. Consequently, large areas need to be included within marine protected areas for effective management and conservation of these special ecosystems in the face of increasing anthropogenic disturbance.

## Introduction

Characterising how different measures of diversity change with scale is fundamental to defining many ecological relationships, such as meta-community assembly and ecological connectivity [1], [2], [3]. Dividing species diversity into different components representing local (α-) and regional (γ-) diversity, and species turnover (β-diversity) has greatly informed our understanding of the processes operating over different spatial or temporal scales [4], [5]. β–diversity has gained considerable value as a conservation tool, by representing either species turnover in space or time, or ecological connectivity as defined by the difference between local diversity and the regional species pool [6], [7], [8], [9]. Thus by characterising the rate of species accumulation from place to place, β–diversity is useful in defining regional-scale diversity and assessing change across environmental and biogeographic gradients [10]. In addition, new techniques for estimating species richness that explicitly incorporate species turnover [11], [12] allow investigations of how different environmental features contribute to shifts in species richness across ecological landscapes [13].

The degree to which habitat or biotic features or dispersal processes control diversity is predicted to vary with space and time. Local gradients in habitat heterogeneity are often positively related to β–diversity [14] and, in seafloor habitats biogenic features can be very important [15]. Thus incorporating fine-scale spatial information on biogenic habitats into the analysis of broad-scale biodiversity relationships becomes important [16], [17], [18]. Over large scales, many studies have focused on relationships between diversity and either latitude or productivity, but general and consistent patterns are elusive. For example, on the seafloor of the Southern Hemisphere there does not appear to be a strong latitudinal gradient in species richness, with high diversity apparent both in temperate systems and in the Antarctic [19]. Relationships between species richness and productivity often vary with taxonomic groups, habitats and scales of sampling [20], [21], with the only generalisation being that productivity-diversity relationships change with spatial scale [17]. These factors emphasize the importance of teasing apart relationships incorporating both local and broad-scale factors [18].

Meta-community theory suggests an important role for β-diversity in the context of ecological connectivity [22]. While meta-community models have yet to fully capture the complexity of natural communities, they do provide some important insights. The difference between the regional species pool at the largest extent of a study (γ-diversity) and the species richness of individual sites (α- diversity) is representative of the connectivity of the ecosystem. In the absence of anthropogenic stressors, locations with high richness relative to the regional species pool can be considered ecologically well connected (low β-diversity). This view of ecological connectivity can complement measures of connectivity for individual species based on the analysis of genetic variation, chemical markers or hydrodynamic dispersal potential (e.g., [23], [24], [25], [26]). Thus, in many systems, β-diversity will influence recovery and resilience within the context of habitat loss, connectivity, and community homogenisation [27], [28].

The potential for diversity patterns to be influenced by site history and environmental factors [29] underlies the need to understand the nature of specific systems [30]. Highly complex biogenic habitats, food limitation, and ice effects are commonly emphasized as important factors affecting Antarctic marine diversity [31], [32], [33]. Short-distance dispersal due to asexual reproduction and the brooding of juveniles are especially common in Antarctic communities [34], [35], although some common species do exhibit long-range dispersal [36], [37], [38] and brooding species can have pan-Antarctic distributions. Nevertheless, in comparison to studies at low latitudes, the pace of reproduction, development, colonization and growth is slow in the Antarctic [39]. The environmental factors most relevant to Antarctic coastal marine ecosystems include low temperature and strong seasonal variation in sea ice cover, light and primary production [40]. Sea ice is a major driver of polar marine ecosystems mainly through its control on primary production processes. At the local scale, sea ice, together with variations in snow cover, influence the underwater light regime and thus strongly affect local primary production [41]. At the regional scale, localised areas of open water (polynyas) generate strong contrasts in food supply [42]. Broader scale gradients in the availability of sunlight for photosynthesis are also strong in the Antarctic. Most studies of key processes influencing Antarctic diversity have not yet attempted to quantitatively tease apart the importance of these factors at different spatial scales.

**Figure 1:**
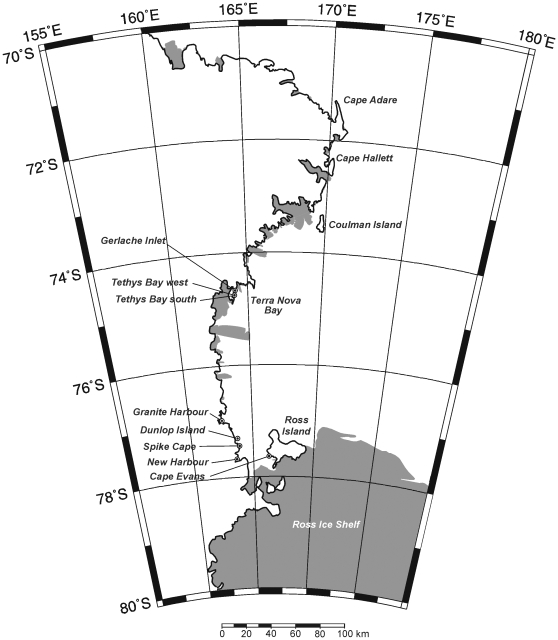
The Victoria Land Coast of the Ross Sea, Antarctica, showing the location of sampling sites.

In this study we use complementary techniques to assess how different processes influence benthic macrofaunal species accumulation, α- and β-diversity, along Antarctica's Ross Sea coast. Specifically, we investigate whether the roles of productivity, habitat and dispersal change with spatial scale by testing predictions that habitat will be most important at the local (e.g., site and location) scale and dispersal will become more important with distance along the coast from McMurdo Sound to Terra Nova Bay (Fig. 1). For each comparison of how distance, habitat and productivity influence species accumulation we develop a null hypothesis: (i) Distance; if all locations were connected equally there would be no sign of increasing species richness with inclusion of more distant locations. This addresses whether all locations are connected equally to the regional species. (ii) Habitat; within-location habitat data will not influence species richness. The null model for both (i) and (ii) is the total species curve [11] which shows an increase in number of species with an increase in number of areas sampled but does not differentiate between areas that are spatially contiguous and does not specifically account for habitat variation. (iii) Productivity; rank ordering locations based on productivity surrogates (e.g., latitude, ice thickness, ice duration, maximum water temp, light at sea floor and sediment Chl a, phaeophytin, % carbon and nitrogen) should not produce a species accumulation curve that is significantly different from a randomized species accumulation curve. We focus on shifts in species richness but also consider changes in species abundance through multivariate variance partitioning to assess the role of these three factors on community structure.

## Results

Surveys of seafloor habitats and macrobenthic communities were conducted at eight locations (Cape Evans, CE; New Harbour, NH; Dunlop Island, DI; Spike Cape, SC; Granite Harbour, GH; Terra Nova Bay West, TNBW; Terra Nova Bay South, TNBS; and Gerlache Inlet, GI; Fig. 1). With the exception of Cape Evans on Ross Island, these locations extend from the western shore of McMurdo Sound to Terra Nova Bay, about half way along the Victoria Land Coast. Although the 4° latitudinal gradient we exploit is short, summer water temperature doubles from McMurdo Sound to Terra Nova Bay (from about −2 to −1°C), and large gradients in sunlight, permanency of ice cover and productivity are encompassed. Our sampling design enabled us to assess variability within locations (based on 3×20 m transects each separated by about 50 m) and between locations ranging from about 3–325 km. A total of about 120 taxa were sampled in 120 cores (70 mm diam., 100 mm deep), emphasizing the diversity of coastal macrofaunal communities in the Ross Sea.

### Species accumulation in relation to distance, habitat and productivity

**Figure 2:**
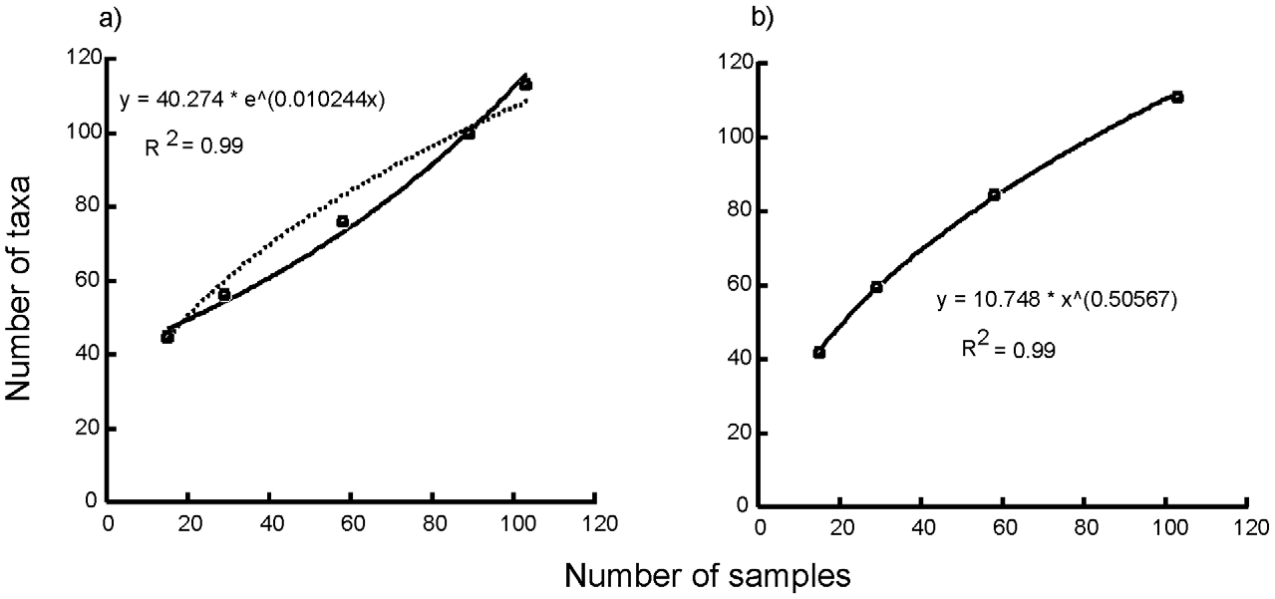
Species accumulation by (a) distance and (b) area. The observed number of taxa (using Mao-Tau) for certain sample sizes, based on accumulation of (a) spatially contiguous samples and (b) random samples, are represented by dots and the curve that best fits the dots by a solid line and equation. To demonstrate the differences between the 2 forms of curves, we show an additional curve for the species accumulation by distance plot (a) (dashed line). This is derived from the response function that was the best fit for species accumulation by area. Samples are 7 cm diam. cores, 10 cm depth.

The randomised species accumulation by area curve was best described by a log-linear model (r^2^ = 0.99, solid line second panel, Fig. 2). In contrast, accumulating species with increasing distance between locations was best described as an exponential model (r^2^ = 0.99, solid line 1st panel), with a log-linear model having a poorer fit (r^2^ = 0.88, dashed line 1st panel). This refutes our null hypothesis and suggests that β–diversity is related to distance, and γ-diversity increases exponentially as sites from further away are sampled.

**Figure 3:**
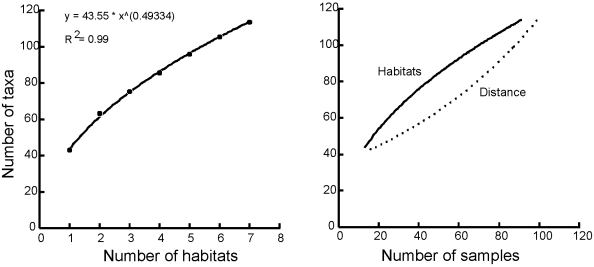
Species accumulation curves by (a) habitats and (b) area. (a) The observed number of taxa (based on Mao-Tau) accumulated over habitats are represented by dots and the curve that best fits the dots by a solid line and equation. (b) Species accumulation curves based on habitats and spatially contiguous samples show different shaped curves. Samples are 7 cm diam. cores, 10 cm depth.

Species richness increased with the number of habitats as a power function, with little sign of an asymptote (Fig. 3). Interestingly, comparison of the observed increase in richness with sample size predicted by accumulating across habitats and distance suggests that habitat diversity is the important driver at small scales, with the distance-based prediction alone clearly underestimating the number of taxa at these scales. Conversely, the distance-based species accumulation curve shows higher accumulation rates at larger scales, suggesting that distance between sites, and thus connectivity, becomes more important at these scales.

**Figure 4:**
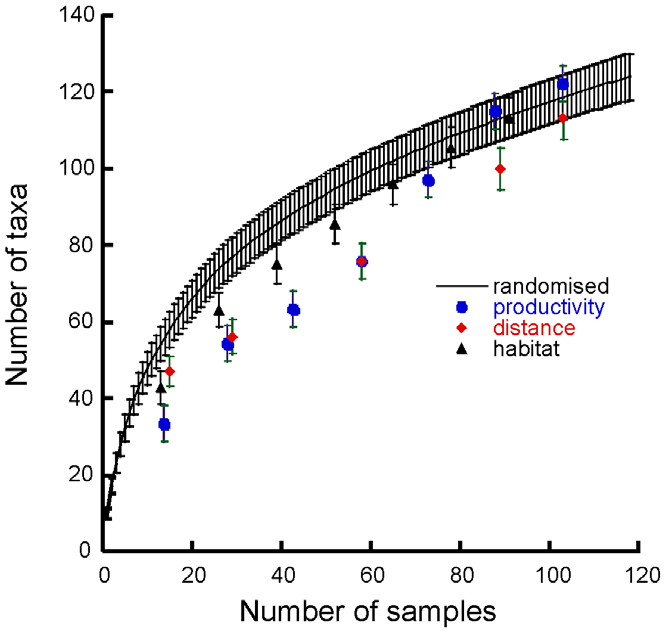
Comparison of species accumulation based on habitat, distance and productivity. The randomized species accumulation curve and associated standard deviation highlight the contrast with the number of species accumulated on the basis of productivity (lowest to highest), distance and number of habitats. Samples are 7 cm diam. cores, 10 cm depth.

The species accumulation curve based on ordering sites from low to high productivity fell outside the 95% confidence intervals for the randomized species accumulation curve, except for the two most productive locations (Fig. 4). Direct comparison of the effects of productivity to those of distance and habitat shows that productivity contributes to regional diversity at spatial scales close to the extent of our study. This analysis also shows the species accumulation curve based on local habitat heterogeneity to most closely track the traditional randomized species accumulation.

### Partitioning

The redundancy analysis (RDA) on which variance partitioning was based explained from 41 to 60% of the variability in macrobenthic community structure, with the amount explained increasing with spatial scale (Table 1). We present the results of our scale-dependent partitioning as a percentage of the explainable fraction. This demonstrates that habitat heterogeneity has a greater control on β-diversity than purely spatial characteristics until samples separated by >70 km are added into the analysis. At the broadest scale purely spatial effects, explained by distance but none of our other explanatory variables, become most important. The effects of productivity surrogates are low and significant only at the three largest scales (<70, <287 <324 km).

**Table 1:** Variance partitioning of based on RDA of Hellinger dissimilarity at different spatial scales.

Scale (km)	Relative importance of explanatory factors	Variance partition (%)			Variance explained by RDA (%)
		Habitat	Productivity surrogate	Purely spatial	
18.5	Habitat > Distance	67	0	33	43
37.0	Habitat > Distance	63	*9*	28	43
70.0	Habitat > Purely spatial and Production	65	18	16	49
287.0	Purely spatial > Production	43	14	43	56
324.0	Purely spatial> Habitat >Production	32	13	55	60

The importance of explanatory factors is represented as a percentage of the variance explained by each RDA. All listed variance % >0 are significant (P<0.05), except for productivity at the 37 km scale.

### Modelling local and regional β-diversity of species richness

Simple regression models with few explanatory variables explain a large proportion of the variation in β-diversity at both local (within locations) and regional scales (Table 2). At the local scale, β-diversity is positively related to habitat variability (Index of Multivariate Dispersion) and mean % C content of the sediment; but negatively related to variability in % C within locations. Regional β-diversity is positively related to the log distance (species accumulating from south to north) and negatively related to mean sediment %N. These results are consistent with our other forms of analysis, reflecting the importance of distance on species connectivity at larger scales and the importance of habitat at smaller scales, with more productive sites having greater site species richness (as per the analysis of species accumulation).

**Table 2:** The influence of explanatory variables on local (sites within locations) and regional β-diversity as revealed by generalized linear models.

Scale	Exp	Source	DF	MS	Parameter estimate	P
Site	0.90	Model	3	3.48		
		Error	4	0.28		
		Total	7	.		
		Intercept	1		3.24	<.0001
		Sediment mean %C	1		0.51	<.0001
		Sediment CV %C	1		−0.99	<.0001
		Habitat variability (IMVD)	1		0.26	0.0859
Regional	0.82	Model	2	262.69		
		Error	5	23.63		
		Total	7	.		
		Intercept	1		83.25	<.0001
		log distance	1		20.36	0.0089
		Sediment mean %N	1		−14.57	0.0252

Models are based on variables standardized to run from 0 to 1. Local β-diversity model used a log-link function and Poisson error structure; regional β-diversity model used an identity link function and normal error structure (Exp  =  ratio of model to total mean square (or deviance), MS  =  mean square or deviance).

## Discussion

Our application of species accumulation models, multivariate variance partitioning and regression all suggest shifts in the relative importance of different processes influencing species accumulation and β–diversity at different spatial scales. These consistencies were apparent both for species presence/absence and abundance data. Three commonly identified factors, distance (Fig. 2), habitat heterogeneity (Fig. 3) and productivity (Fig. 4), each affected both the rate of species accumulation along the Victoria Land Coast and variation in species composition within locations (Table 2). It would have been surprising if we had not detected a scale-dependent response, especially in β–diversity, as species turnover should exhibit positive spatial autocorrelation [43]. However, our results highlight a key problem with tests of macroecological biodiversity relationships, namely, the search for simple causative relationships that function across scales is likely to be less insightful than acknowledging the potential for a range of variables to be involved in driving emergent patterns.

One interpretation of high β–diversity is that it reflects poor connectivity between meta-communities, assuming that localised disturbance events have not led to local extinction. Locations that are species rich relative to the regional species pool are well connected and are likely to exhibit high spatial variability in community composition within n individual location or site. Our combined analyses show the important influence of distance between sites on β-diversity and overall species richness (Fig. 2, Tables 1 and 2). Distance can act as a surrogate for many environmental variables but the species accumulation models that accumulate species based on inter-location distance emphasize connectivity to the regional species pool. Distance between our eight locations, and thus connectivity and dispersal, becomes exponentially important at larger scales, implying that as we move northwards connectivity between locations increases. This finding is consistent with the oceanographic information available for this area of the Victoria Land Coast and Ross Island [44]. Water moves to the south past Cape Evans to flow under the Ross Ice Shelf, with some water returning into McMurdo Sound and in a northerly direction along the Victoria Land Coast. Secondly, westerly and northerly water flow increases in strength to the north. Limited hydrodynamic connectivity suggests a potential for shifts in resilience and slow recovery from disturbance. These effects on ecosystem dynamics have important conservation and management implications for a region faced with increased commercial fishing, tourism and climate change.

Analytical approaches that move away from defining the right scale to address variation in biodiversity across scales will be particularly important in ecosystems such as coastal Antarctica with high biogenic habitat heterogeneity and little history of human impact. Habitat diversity positively effects β-diversity in our study, increasing regional species richness across the extent of our sampling (Fig. 4). Our scale-dependent multivariate variance partitioning and our generalized linear models also illustrate the importance of habitat heterogeneity in contributing to species turnover, both within locations and as the dominant source of variability in community composition up to scales of 70 km. Clearly, habitat effects are not restricted to small spatial scales. Similar results in very different ecological systems emphasize that while habitat effects may be focused at small-scales, they nevertheless can also function over broad scales [45].

Gathering information on the productivity of different locations, where production sources may vary between sites and times, is difficult in coastal Antarctica. Nevertheless, the productivity ranking of our locations supported the prediction of a positive influence of productivity on species richness. These positive effects seem to be restricted to the more productive sites, rather than reflecting a more linear relationship. The positive role of productivity in affecting the benthos of the high Antarctic is consistent with [42], thus extending these patterns north from McMurdo Sound to the mid-Victoria Land Coast (Terra Nova Bay). Our parsimonious generalized linear models also emphasize the role of productivity on β-diversity both at local and regional scales. Local effects on β-diversity were strong, with average sediment %C content increasing β-diversity within locations. Despite the limitation of using sediment %C as a surrogate for productivity at these latitudes where recycling is slow, this effect was ameliorated to some extent by variation in %C within locations. At the regional scale sediment %N content became an important factor positively influencing β-diversity. This shift in the importance of sediment carbon vs nitrogen from local to regional scales is interesting, and may reflect a change from an importance of food quantity and patchiness at local scales, to food quality at regional scales.

Our approach of building species accumulation curves to investigate the potential influence of a specific factor and assessing differences to null models of more randomly assembled communities, provides a new way of teasing apart diversity relationships (Figs. 2 and 3). It has proven effective despite a relatively small number of samples, albeit collected across strong environmental gradients. Overall macrofaunal richness was high, with 120 taxa from 120 cores, and variation in richness between locations of about 40 taxa. Species accumulation with distance or habitat diversity was not well explained by linear-logarithmic relationships, which are suggested to be a better data fit when β–diversity is high at small spatial scales and richness is low [2]. Instead, species accumulation with habitat diversity was best described by a power function, as β–diversity continued to increase with increasing habitat diversity. Importantly, species accumulation with distance was best explained by an exponential curve, emphasizing that β–diversity has its strongest effects on increasing the size of the regional species pool over large scales. This is supported by Fig. 4 which shows differences in the rates of species accumulation based on area (random), distance, habitat and productivity.

Connectivity across landscapes is increasingly recognised as important for the conservation and management of biodiversity [46], [47]. The metacommunity concept that recovery at the patch scale is linked by dispersal potential to the regional species pool highlights the importance of the environmental setting and the potential for cumulative impacts [3], [48], [49]. As our view of the openness of marine communities' changes and knowledge of the poor dispersal ability of many benthic species and the threat of disturbance to seafloor habitats increases so the conservation of pristine habitats must become more proactive. In the context of Antarctic coastal benthic communities, the slow rates of recovery, high ratio of poor dispersing and brooding species, high biogenic habitat heterogeneity and spatio-temporal variability in primary production become especially important considerations for conservation. Our results demonstrate that locations along the Victoria Land Coast are not equally connected. This highlights the importance of enshrining large areas within Marine Protected Areas to ensure they are able to be self-sustaining in the face of increases in the disturbance regime, despite the pan-Antarctic distribution of many common macrofaunal species.

## Materials and Methods

At each sampling location (Cape Evans, New Harbour, Dunlop Island, Spike Cape, Granite Harbour, Terra Nova Bay West, Terra Nova Bay South and Gerlache Inlet) we dived at 3 sites through holes in the sea ice (about 50 m apart). Water depth varied from 15–25 m, which was below the main disturbance effects of anchor ice and fast ice grounding. Surveys were conducted over different years in late-spring to mid-summer (October-January, Appendix S1). On the seafloor at each site we laid a shore parallel 20 m transect and marked the position of 5 random points with numbered metal pegs. At each point we collected one core (70 mm diameter, 100 mm deep) for macrofauna and two cores (26 mm diameter, 50 mm deep) to determine sediment % C and N, and organic and chlorophyll *a* (Chl *a*) content, respectively. To describe the habitat around each core, the transect tape and peg markers were videoed using a diver-held digital video camera, with the camera lens perpendicular to the seafloor at fixed heights of 70 cm and 40 cm above the bottom. We used two heights for videoing to account for differences in the density of habitat features between sites, and we scaled features to numbers or percent cover m^−2^ [50]. We collected information on the thickness and permanency of sea ice, snow cover, current velocity and light transmission to the seafloor at each location (Appendix S1).

Habitat was characterised from a 1.5 m length of video frame grabs centred on each core location (using a Sony DVBK 2000E V1.00, pixel resolution was 1.7 mm). Preliminary analysis of the video transects indicated this was the best scale to describe habitat associated with each core [50]. Features contributing to habitat structure on the seafloor (e.g., sediment characteristics and sedentary epifauna) were quantified and dominant habitat categories were determined for each core. Eight common habitat types were defined for this analysis: *Phyllophora* (macroalgae) and rock; sponges and rock; sponges and scallops; scallops; rock and sand; rock and boulders; cobbles and pebbles; and sand. Each site had at least two habitat types and most habitat types occurred within at least three sites. There was no latitudinal progression in habitat types. All habitat variables were standardized to run between 0 and 100 before analysis. Habitat diversity was defined by the number of habitats occurring at a site and the index of multivariate dispersion of habitat features over the site (IMVD [51]).

Core samples were sieved (500 µm mesh), and then preserved in 70% isopropanol and 0.1% Rose Bengal in seawater. In the laboratory, macrofauna were sorted, and identified to the lowest taxonomic level possible. Sediment from one small core (top 5 cm) was homogenised and sub-sampled for analysis of chlorophyll *a* (Chl *a*) and organic content. Sediment from the other small core (top 0.5 cm) was analyzed to determine % carbon (C) and % nitrogen (N) content. Chl *a* was extracted from freeze dried sediments by boiling in 90% ethanol. The extract was measured spectrophotometrically, and an acidification step was included to separate degradation products (phaeophytin) from Chl *a* [52]. Organic content was determined by drying the sediment at 60°C for 48 h, followed by combustion at 400°C for 5.5 h. Sediment % C and N were determined on freeze-dried sediment samples collected for stable isotope analysis; this involved an acidification step to remove carbonates from the sediments (see [53]).

### Ethics Statement

In compliance with the New Zealand Antarctic Marine Living Resources Act 1981, all field work was conducted under permit from the New Zealand Ministry of Foreign Affairs and Trade (AMLR permit # AMLR06/002/Cummings and Thrush/K082; AMLR08/003/Cummings/K082). Field work in Terra Nova Bay was also conducted under a sampling permit for the Antarctic Specially Protected Area (ASPA) under Part IV, Section 28, entry to ASPA 161 Terra Nova Bay, Ross Sea. Preserved macrofaunal organisms returned to New Zealand for identification and enumeration entered the country under “Permit to Import Restricted Biological Products of Animal Origin” issued by the New Zealand Ministry of Agriculture and Forestry (2002016395; 2006029377; 2008035235).

### Species accumulation curves

To determine macrofauna species richness at each location and across the region, a variety of species accumulation curves were generated [54]. Comparison of species accumulation curves based on increasing area sampled (Mao Tau) or the increasing number of individuals' sampled (Coleman rarefaction) showed no differences, indicating abundance was not strongly influencing species accumulation. While randomization processes are commonly used in the construction of species accumulation curves to simply estimate species richness [54], if the aim of the study is to determine the importance of specific processing in contributing to species richness then it is more informative to construct the species accumulation based on variation in contributing factors [13]. The analytical approach developed by [11] enabled us to construct distance-, habitat- and productivity-based total species curves. Distance-based curves were constructed using locations grouped into <18.5 km, <37 km, <70 km, <287 km and <324 km distance classes. Habitat-based curves were calculated as described in [13] at the same scales. Productivity-based curves were conducted at the location scale only, because of our reliance on productivity surrogate variables (latitude, ice thickness, ice duration, maximum water temp and light at sea floor). We used these variables, plus data on sediment Chl *a*, phaeophytin, % C and N to rank locations from low to high productivity (i.e., NH, SC, DI, GH, TBW, CE, TBS, GI) and then constructed curves based on NH alone, then NH and SC, then NH, SC and DI, etc.

### Regression models

To further understand the relative importance of habitat heterogeneity, distance and productivity in influencing β-diversity at local (within location) and regional (between locations) scales, we developed generalized linear models (GLzM's) that identified important predictors from the following suite of variables: distance north of the southernmost site (NH); number of habitats, habitat IMVD, ice thickness and duration, snow cover, maximum water temperature, and mean and coefficient of variation of Chl *a*, Chl *a* + phaeopigments, organic content, % C and % N. We also tried replacing the variables representing productivity with the productivity ranking and using log, exponential, polynomial and power transformations to incorporate non-linearity. Local α-diversity in species richness was defined as: total number of species observed at a site minus the average number of species observed in core replicates at that site. Regional β-diversity was defined as: total number of species observed over the entire study minus the total number of species observed at a location.

The appropriate error structure to be used in building the GLzMs was determined using visual inspection of half-normal plots of residuals and plots of residuals vs predicted values. This resulted in the use of a log-link function and Poisson error structure to model local β-diversity and an identity link function and normal error structure to model regional β-diversity. Parsimonious models were developed by backwards elimination with a variable exit criteria of P = 0.15 [55]. Colinearity diagnostics were examined for all GzLM analyses, to ensure that highly correlated environmental variables were not included in the final model [56]. If over dispersion was indicated for Poisson error structures (Pearson χ2/d.f.>3), quasi-likelihood estimation was used.

### Diversity partitioning

To define whether presence/absence patterns observed in the species accumulation curves were generalisable to measures of β-diversity based on species abundance we used variance partitioning procedures [45], [57]. Redundancy analysis (RDA) of Hellinger transformed data was conducted on datasets representing cores within <18.5 km, <37 km, <70 km, <287 km and <324 km distances classes and the amount of variability explained by habitat, productivity, habitat related productivity and purely spatial factors were determined. Habitat was represented by 7 dummy variables and productivity by the productivity rank (note that the effect of productivity could not be determined until a spatial scale of 70 km as the gradient of effects was insufficient at smaller scales). Spatial factors were dummy variables representing the cluster of sites formed at different distances and, in the full data set, distance north from the southernmost site. In this context, distance between sites represents a surrogate for connectivity and unmeasured environmental variables unrelated to productivity that change along the coast. Due to data restrictions we were unable to separate the effects of habitat heterogeneity and purely spatial variance at the within-site scale.

## Supporting Information

Appendix S1 Summary of environmental factors at each location/site.
(0.07 MB DOC)
