# Supplementary material for: β-Diversity and Species Accumulation in Antarctic Coastal Benthos: Influence of Habitat, Distance and Productivity on Ecological Connectivity
Source: PLoS One. 2010 Jul 30;5(7):e11899. doi: 10.1371/journal.pone.0011899 (PMC2912761; doi:10.1371/journal.pone.0011899)
Supplement: Appendix S1 — Summary of environmental factors at each location/site. (0.07 MB DOC) [file pone.0011899.s001.doc]

**APPENDIX 1**

Summary of environmental factors at each location/site.

| Location | Site | Depth (m) | Lat (^o^S) | Long (^o^E) | Date sampled | Ice thickness (m) | Snow cover (m) | Ice duration (months p.a.) | Current speed (mean + SD) | % incident light at seafloor | Water temp (max) (^o^C) |
| --- | --- | --- | --- | --- | --- | --- | --- | --- | --- | --- | --- |
| NH | 1 | 24 | 77° 34.578 | 163° 31.668 | Nov-01 | 3.5 | 0 | 12 | 2.6 ± 0.8 | 0.1 | -1.92 |
|  | 2 | 19 | 77° 34.555 | 163° 31.279 | Nov-01 | 3.5 | 0 | 12 |  |  |  |
|  | 3 | 22 | 77° 34.610 | 163° 31.895 | Nov-01 | 3.5 | 0 | 12 |  |  |  |
| CE | 1 | 21 | 77° 38.072 | 166° 24.883 | Nov-01 | 2.5 | 0 | 10 to 11 | 3.5 ± 1.3 | 0.1 | -1.92 |
|  | 2 | 19.5 | 77° 38.072 | 166° 24.993 | Nov-01 | 2.5 | 0 | 10 to 11 |  |  |  |
|  | 3 | 19 | 77° 38.095 | 166° 24.843 | Nov-01 | 2.5 | 0 | 10 to 11 |  |  |  |
| DI | 1 | 19 | 77° 14.161 | 163° 27.940 | Nov-02 | 2.6 | 0.1 | 10 to 11 | 3.8+2.6 | nr | -1.92 |
|  | 2 | 21 | 77° 14.176 | 163° 27.997 | Nov-02 | 2.6 | 0.1 | 10 to 11 |  |  |  |
|  | 3 | 15.5 | 77° 14.141 | 163° 27.917 | Nov-02 | 2.6 | 0.1 | 10 to 11 |  |  |  |
| SC | 1 | 18.5 | 77° 18.024 | 163° 33.935 | Nov-02 | 2.4 | 0.15 | 10 to 11 | 2.6+1.3 | nr | -1.92 |
|  | 2 | 20 | 77° 18.040 | 163° 33.880 | Nov-02 | 2.4 | 0.15 | 10 to 11 |  |  |  |
|  | 3 | 14.8 | 77° 18.050 | 163° 33.958 | Nov-02 | 2.4 | 0.15 | 10 to 11 |  |  |  |
| GH | 1 | 17.2 | 77° 01.013 | 162° 52.693 | Nov-07 | 2.1 | 0 | 10 to 11 | 3.0 + 1.85 | 0.02 | -1.92 |
|  | 2 | 18.8 | 77° 00.984 | 162° 52.644 | Nov-07 | 2.1 | 0 | 10 to 11 |  |  |  |
|  | 3 | 19.8 | 77° 00.955 | 162° 52.575 | Nov-07 | 2.1 | 0 | 10 to 11 |  |  |  |
| TBW | 1 | 22 | 74 40.859 | 164 03.494 | Jan-03 | 2 | 0 | 9 | nr | nr | nr |
|  | 2 | 23.9 | 74 40.826 | 164 03.569 | Jan-03 | 2 | 0 | 9 |  |  |  |
|  | 3 | 23.8 | 74 40.791 | 164 03.624 | Jan-03 | 2 | 0 | 9 |  |  |  |
| TBS | 1 | 21 | 74° 41.398 | 164° 06.787 | Nov-06 | 2.8 | 1-1.5 | 8 | 1.83 + 1.13 | 0.21 | -1.89 |
|  | 2 | 21.5 | 74° 41.396 | 164° 06.661 | Nov-06 | 2.8 | 1-1.5 | 8 |  |  |  |
|  | 3 | 21.0 | 74° 41.395 | 164° 06.529 | Nov-06 | 2.8 | 1-1.5 | 8 |  |  |  |
| GI | 1 | 20.7 | 74° 40.265 | 164° 03.698 | Nov-06 | 2.8 | 0 | 8 | 1.99 + 1.21 | 0.1 | -1.90 |
|  | 2 | 21.5 | 74° 40.238 | 164° 03.674 | Nov-06 | 2.8 | 0 | 8 |  |  |  |
|  | 3 | 16.7 | 74° 40.209 | 164° 03.624 | Nov-06 | 2.8 | 0 | 8 |  |  |  |

Locations: NH = New Harbour; CE = Cape Evans; DI – Dunlop Island; SC = Spike Cape; GI = Gerlache Inlet; TBS = Terra Nova Bay South; GH = Granite Harbour; TBW = Terra Nova Bay West

Current velocities and water temperatures were measured by an S4 or Anderra current meter deployed 4 m above the seabed for the duration of our visit (at least 3 days). Percentage incident light was calculated from 5 replicate measurements of photosynthetically available radiation (PAR) made both above the sea ice and at the seafloor, using a LiCor Li190SA quantum sensor for incident irradiance (background irradiance above the ice) and a Li192SB for underwater irradiance attached to a Li-1000 logger. Permanency of sea ice was estimated using a combination of examination of satellite images, available literature, and personal observations.
